# Supplementary material for: Characterization of nanoparticles combining polyamine detection with photodynamic therapy
Source: Commun Biol. 2021 Jul 1;4:803. doi: 10.1038/s42003-021-02317-5 (PMC8249666; doi:10.1038/s42003-021-02317-5)
Supplement: Supplementary file 2 — Supplementary Information [file 42003_2021_2317_MOESM2_ESM.pdf]

Supplementary Information

Characterization of nanoparticles combining polyamine detection with photodynamic therapy

Wenting Li,[1] Lingyun Wang,*[1] Tianlei Sun,[2] Hao Tang,[2] Brian Bui[3], Derong Cao,[1] Ruibing Wang,*[2] and Wei Chen*[3]

[1]Key Laboratory of Functional Molecular Engineering of Guangdong Province, School of Chemistry and Chemical Engineering, South China University of Technology, Guangzhou, 510641, China. E-mail: lingyun@scut.edu.cn; Fax: +86 20 87110245; Tel: +86 20 87110245

[2]State Key Laboratory of Quality Research in Chinese Medicine, Institute of Chinese Medical Sciences, University of Macau, Taipa, Macau SAR, China. Email: rwang@um.edu.mo.; Tel: +853-8822-4689

[3]Department of Physics, University of Texas at Arlington, Arlington, TX 76019, USA, Email: weichen@uta.edu

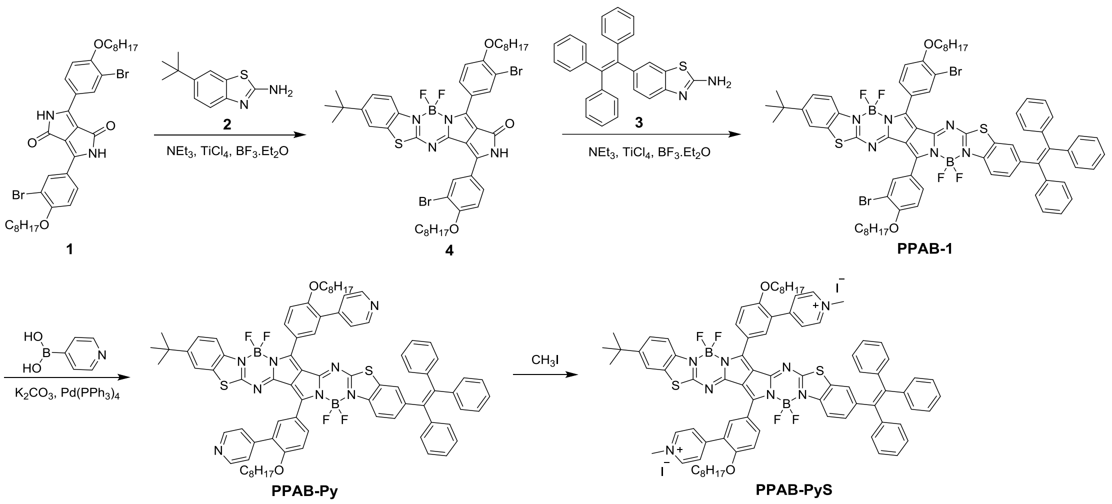

Supplementary Figure 1. The synthetic routes of **PPAB-PyS**.

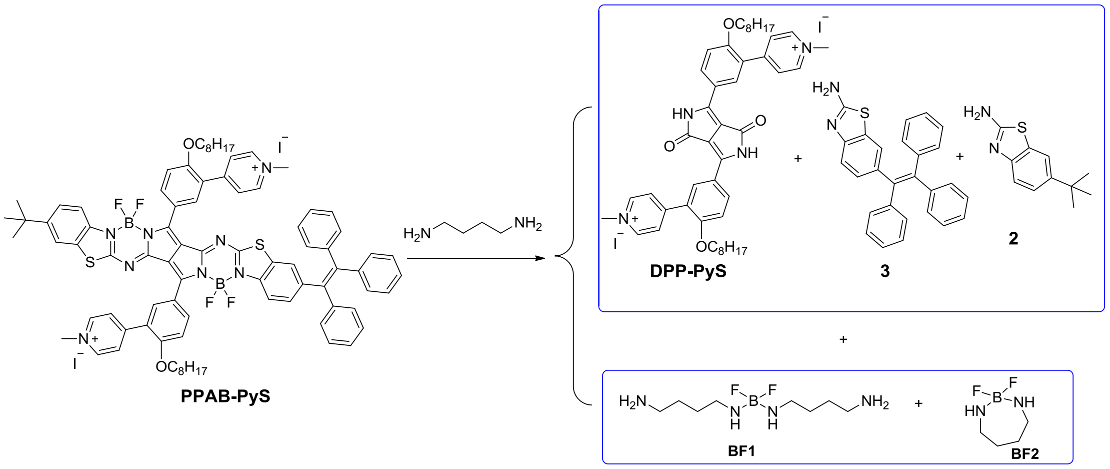

Supplementary Figure 2. The possible reaction mechanism between **PPAB-PyS** and putrescine.

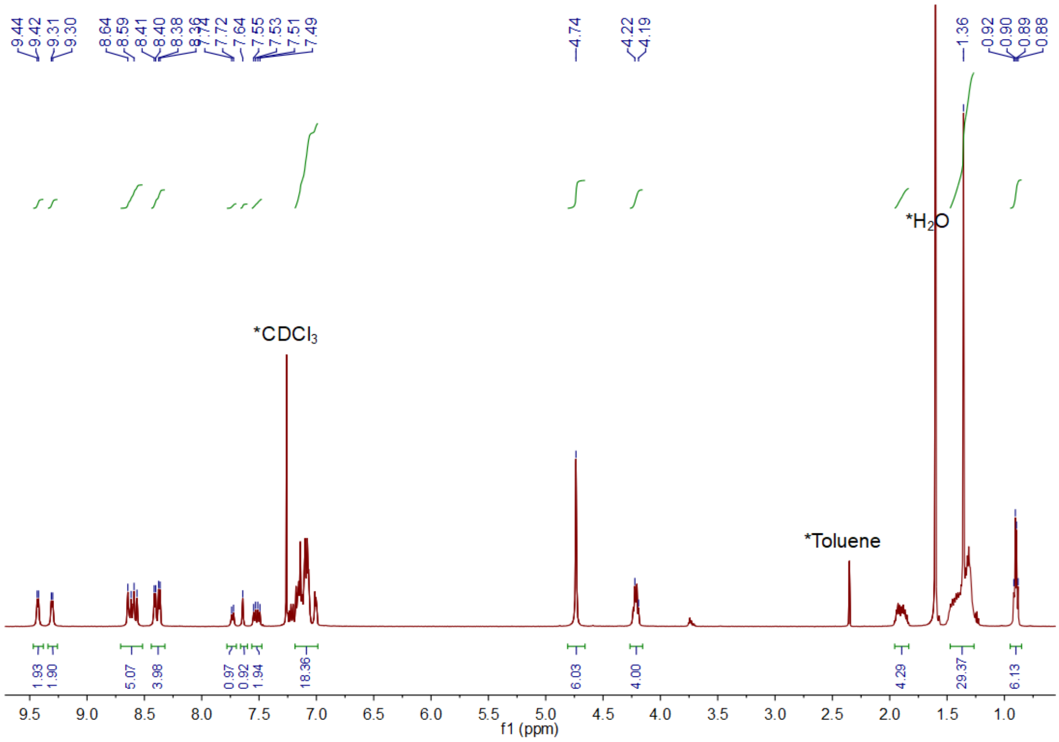

Supplementary Figure 3 ^{1}H NMR spectrum of **PPAB-PyS**in $CDCl_3$.

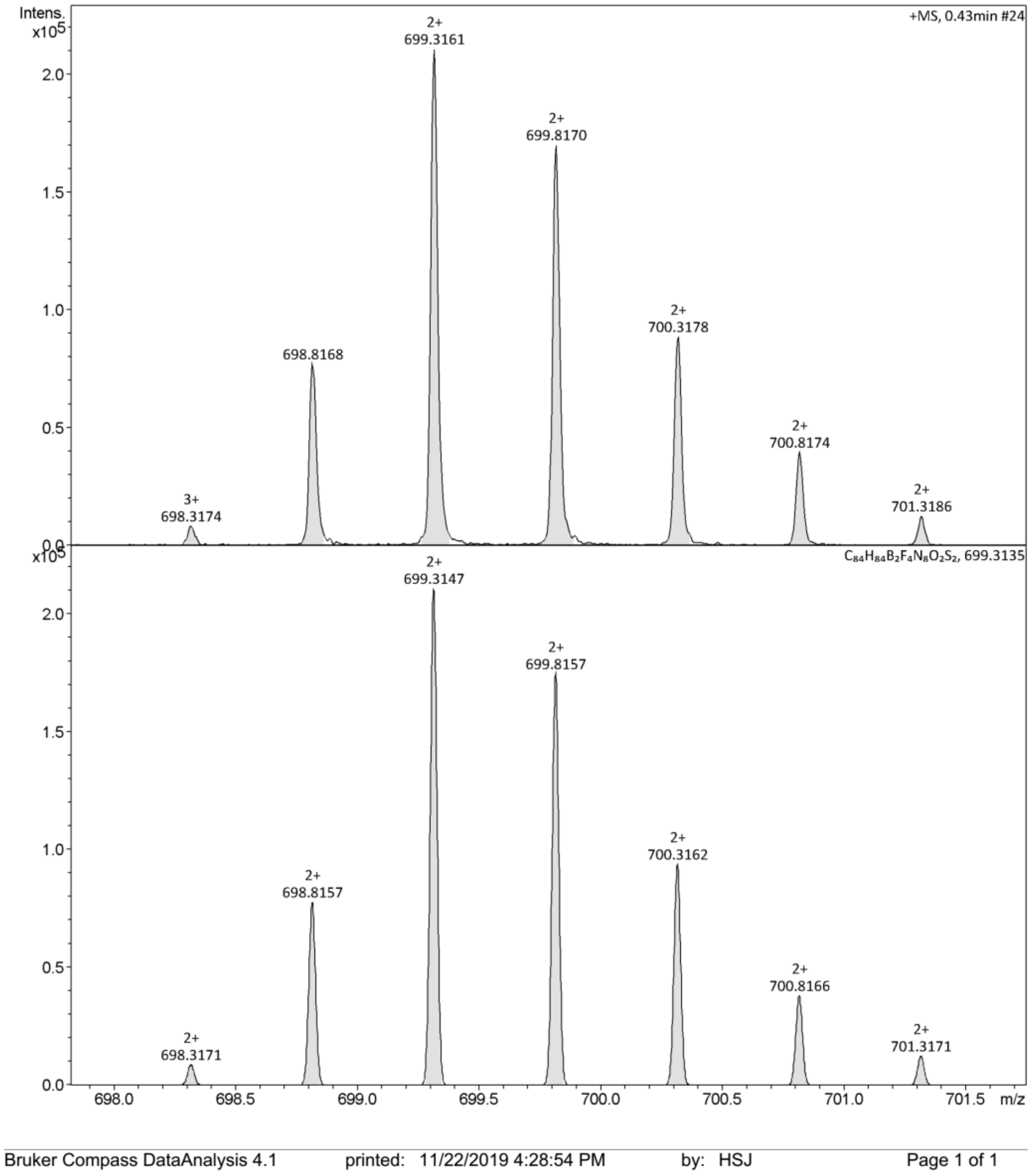

Supplementary Figure 4. HRMS spectrum of **PPAB-PyS**.

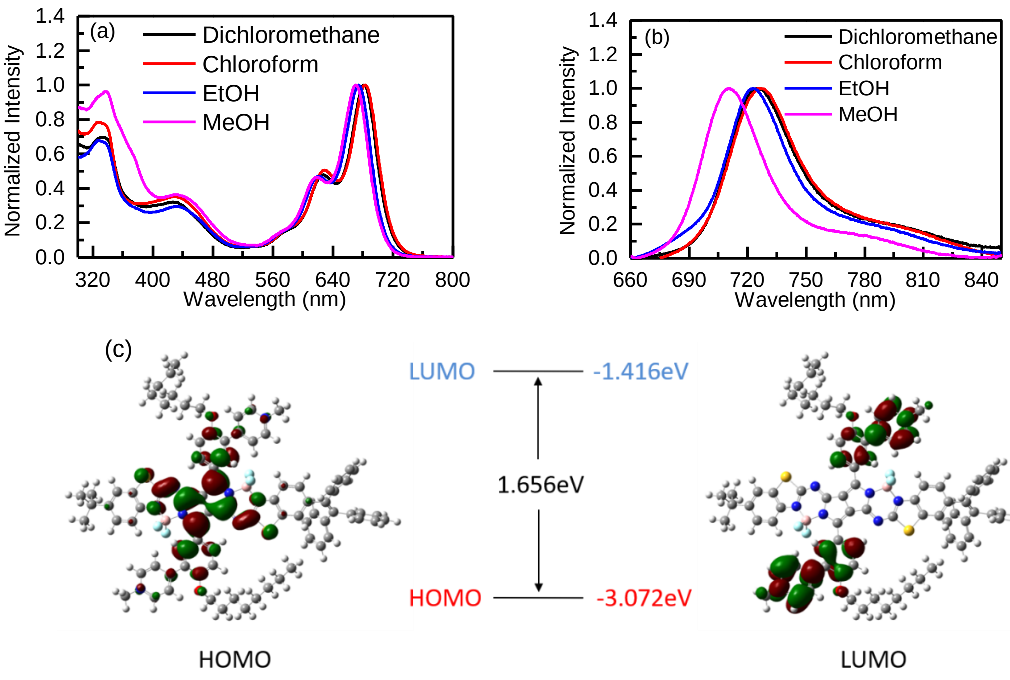

Supplementary Figure 5.(a) UV-vis and (b) emission spectra of **PPAB-PyS** (10 μM) in different solvents. (c) Optimized molecular conformation and molecular orbital amplitude plots ofHOMO and LUMO energy levels of **PPAB-PyS**.

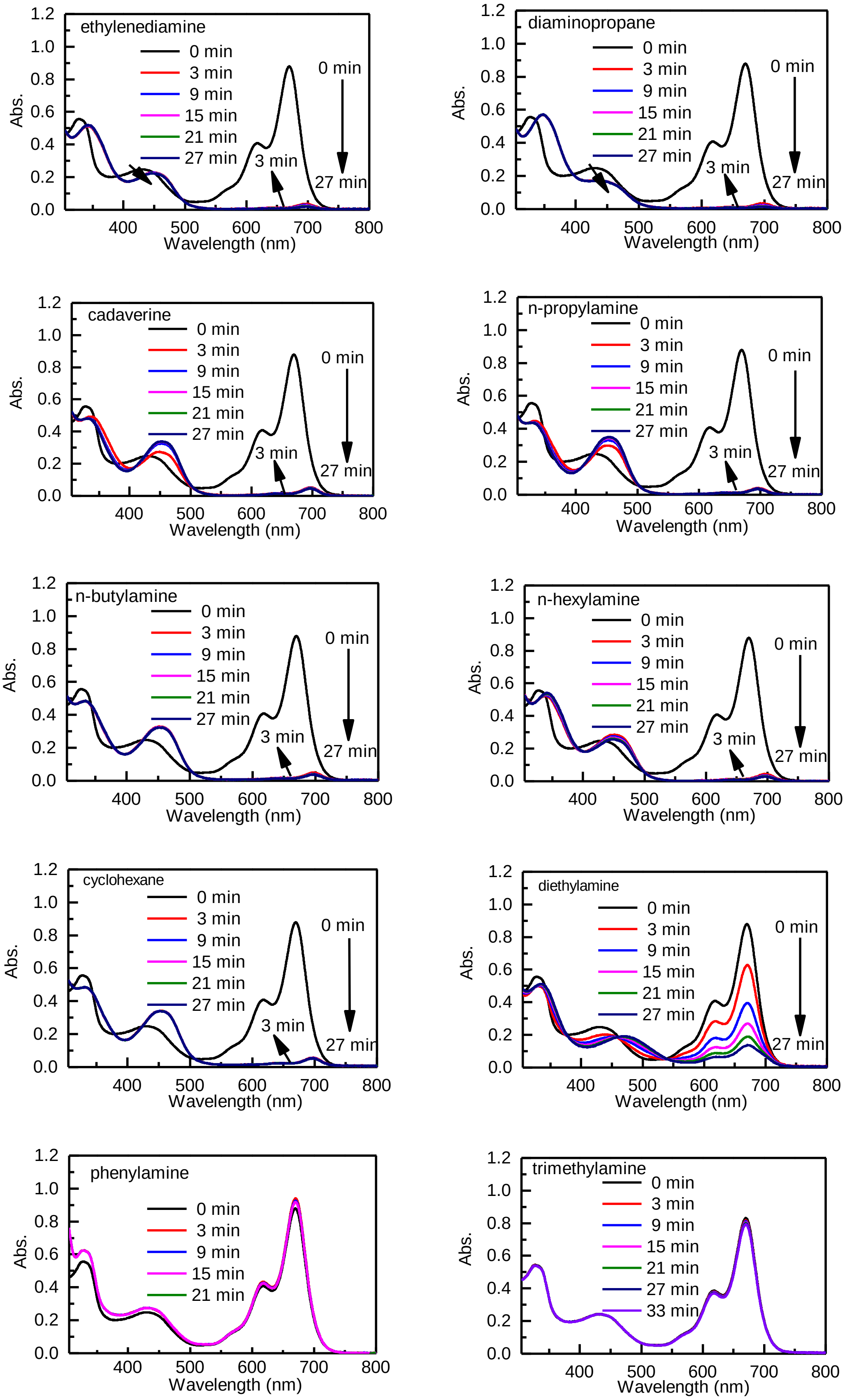
ethylenediamine
0 min
3 min
9 min
15 min
21 min
27 min
diaminopropane
cadaverine
n-propylamine
n-butylamine
n-hexylamine
cyclohexane
diethylamine
phenylamine
trimethylamine
33 min
Abs.
Wavelength (nm)

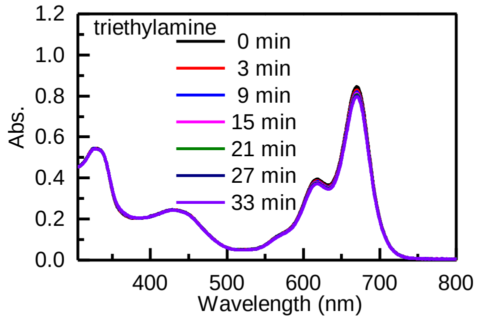

Supplementary Figure 6. Time-dependent UV-vis absorption spectra of **PPAB-PyS**(10 μM) in MeCN in presence of different amine (40 equiv.) at 25 °C.

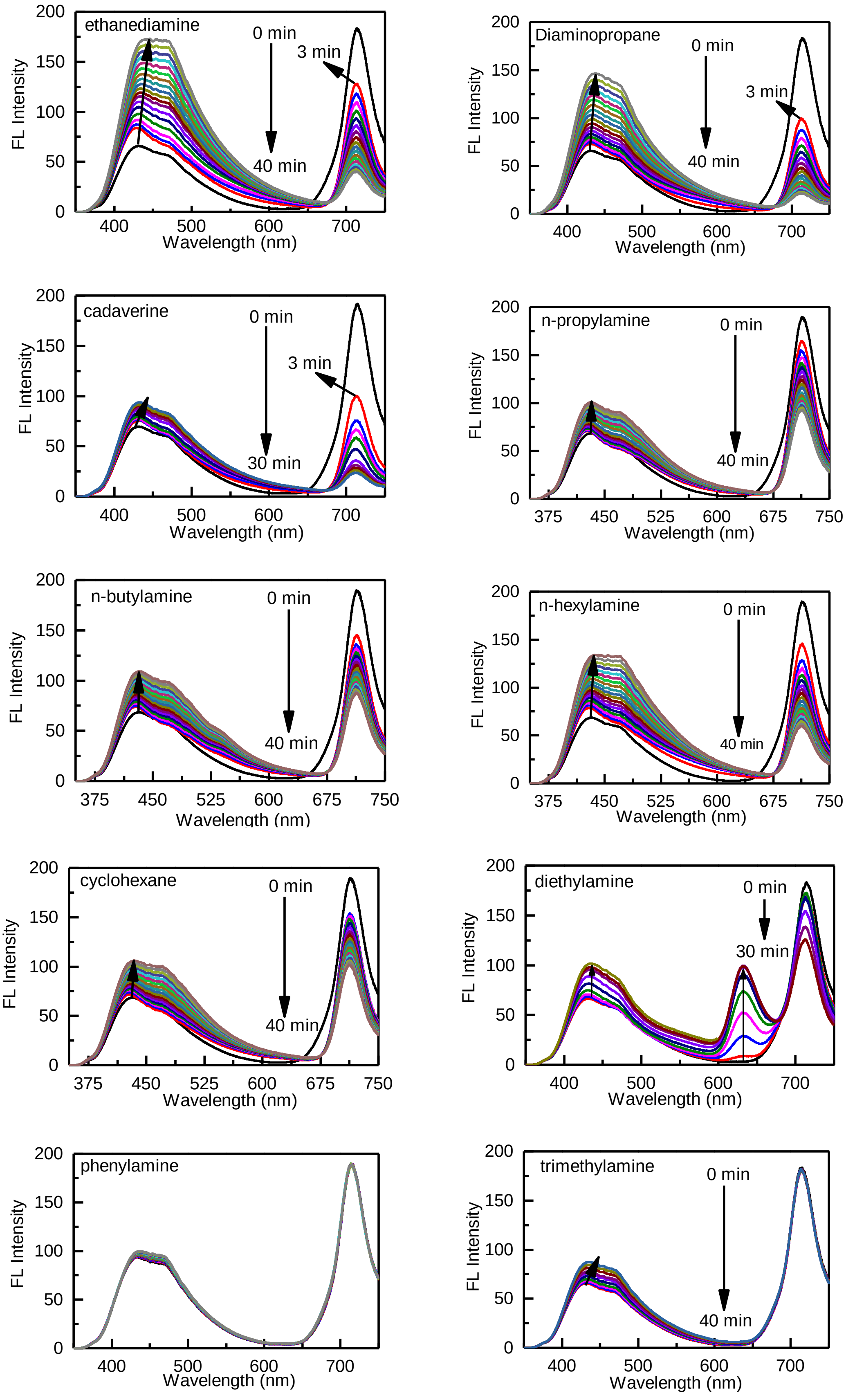
ethanediamine
0 min
3 min
40 min
Diaminopropane
0 min
3 min
40 min
cadaverine
0 min
3 min
30 min
n-propylamine
0 min
40 min
n-butylamine
0 min
40 min
n-hexylamine
0 min
40 min
cyclohexane
0 min
40 min
diethylamine
0 min
30 min
phenylamine
trimethylamine
0 min
40 min
FL Intensity
Wavelength (nm)

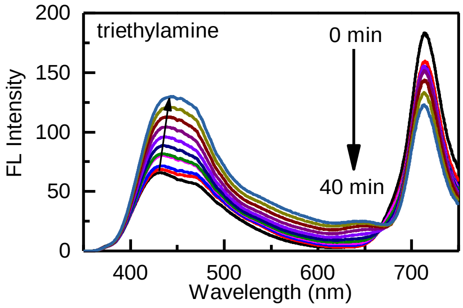

Supplementary Figure 7. Time-dependent emission spectra of **PPAB-PyS**(10 μM) in MeCN in presence of different amine (40 equiv.) at 25 °C.

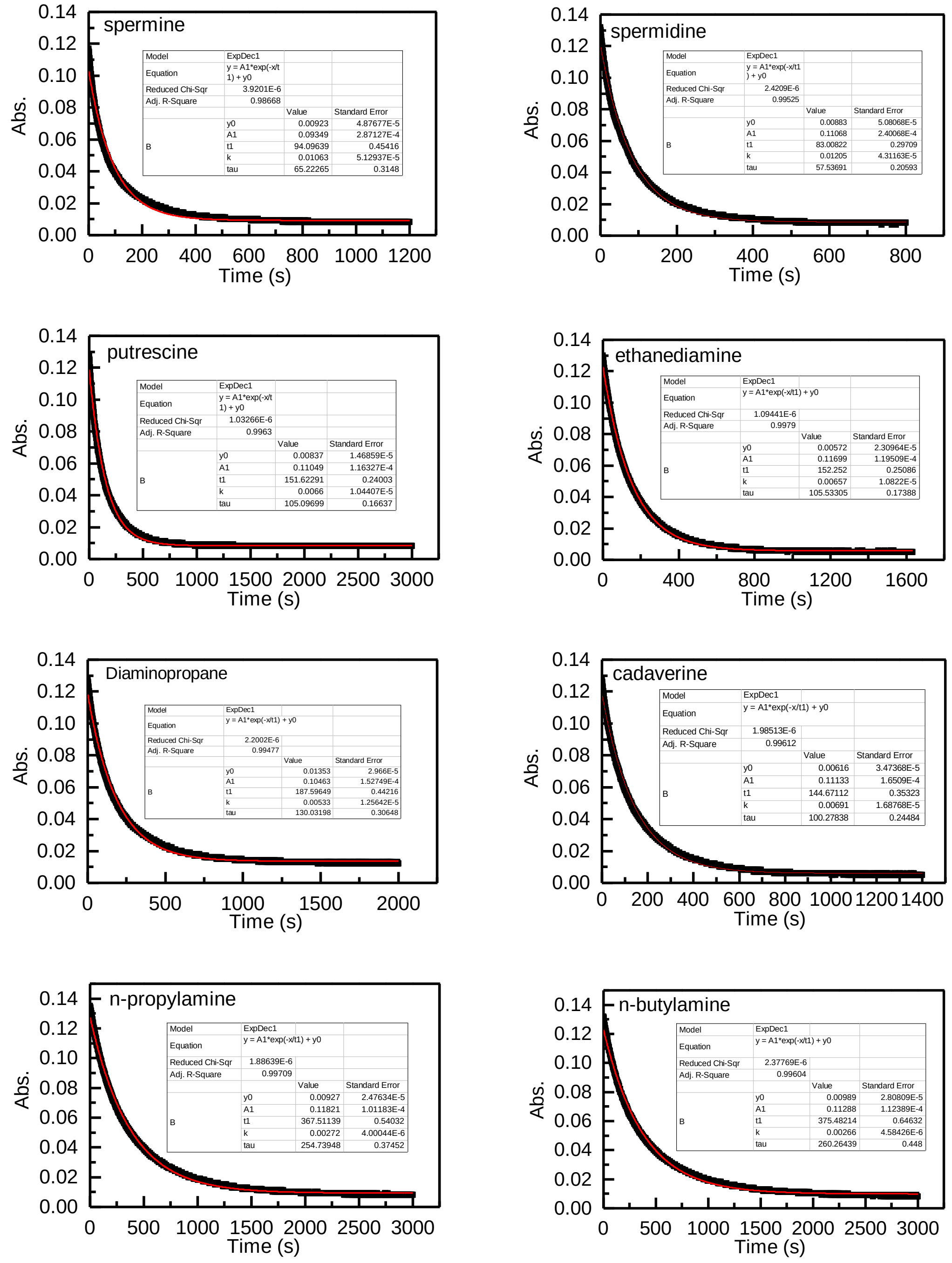

Supplementary Figure 8. The kinetics reaction of**PPAB-PyS** (1.25μM)inMeCN in presence of different amine(4 μM) at 22 °C.

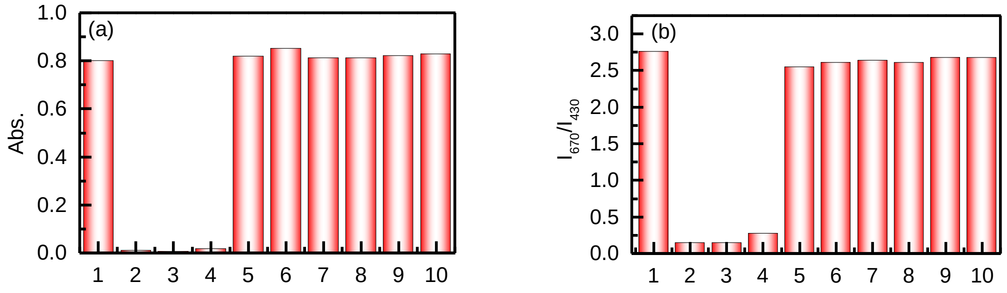

Supplementary Figure 9. (a) The absorption of 680 nm and I_{710}/I_{430} ration for depicting selectivity of **PPAB-PyS**(10 µM) toward polyamine (40 equiv.) and various other analytes (100 equiv.)for 30 min at 25 ^{o}C.The polyamine and various other analytes as followed: (1) blank, (2) Spermidine, (3) Spermidine, (4) Putrescine, (5) H_2O_2, (5) NO_3^-, (6) NO_2^-, (7) Na^+, (8) K^+, (9) Glutathione.

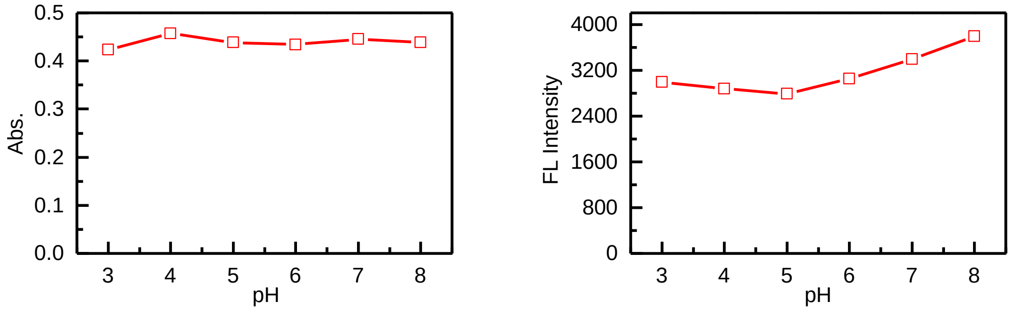

Supplementary Figure 10. Effect of the pH on the (a) absorption (A_{695} nm) and (b) emission(I_{456}) of **PPAB-PyS**(10 µM) in DMSO–PBS buffer solution (MeCN/PBS = 1: 200, v/v).

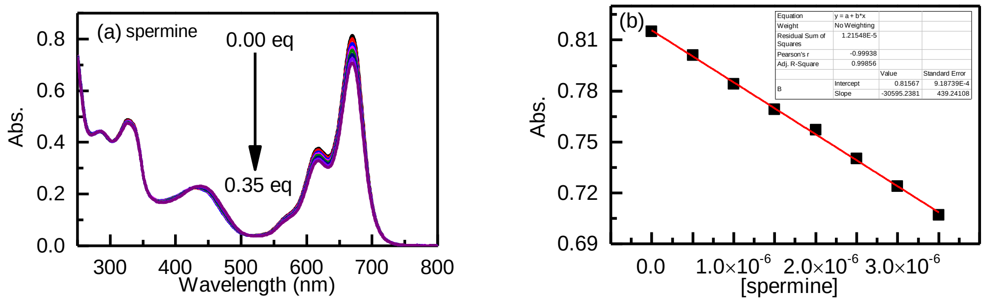

Linear Equation: $Y=-30595.23X+0.81567$ $R = 0.99938$

$S = 3.05 \times 10^4$ $\delta = \sqrt{\frac{\sum(A_0 - A_1)^2}{N-1}} = 0.001972539$ ($N = 11$) $K = 3$

$LOD = K \times \delta / S = 3 \times 0.00197 / 3.05 \times 10^4 = 0.193$ μM

A_0 is the absorbance intensity of **PPAB-PyS** at 670 nm; A_1 is the average of the A_0.

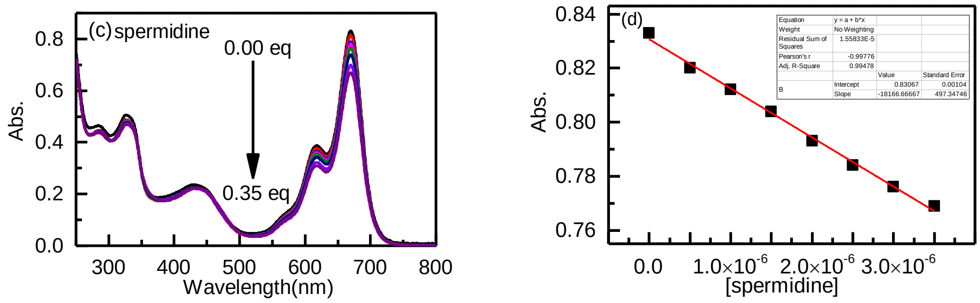

Linear Equation: $Y=-18166X+0.8306$ $R = 0.99776$

$S = 1.81 \times 10^4$ $\delta = \sqrt{\frac{\sum(A_0 - A_1)^2}{N-1}} = 0.001972539$ ($N = 11$) $K = 3$

$LOD = K \times \delta / S = 3 \times 0.00197 / 1.81 \times 10^4 = 0.326$ μM

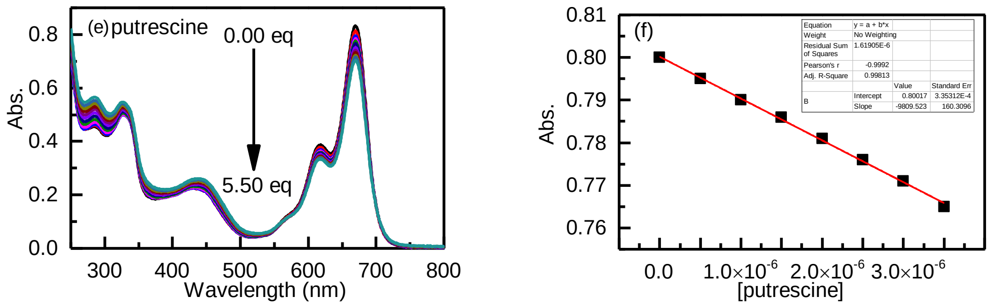

Linear Equation: $Y=-9809X+0.80017$ $R = 0.9992$

$S = 9.81 \times 10^3$ $\delta = \sqrt{\frac{\sum(A_0 - A_1)^2}{N-1}} = 0.001972539$ ($N = 11$) $K = 3$

$LOD = K \times \delta / S = 3 \times 0.00197 / 9.81 \times 10^3 = 0.602$ μM

Supplementary Figure 11. (a-f) Concentration-dependent UV-vis spectra of**PPAB-PyS** (10 µM) in MeCN in presence of (a) spermine, (c) spermidine and (e) putrescineat 25 ºC, each solution was mixed and left for 5 min. The linear relationship between (b)spermine, (d) spermidine and (f) putrescine and absorption of 670 nm of **PPAB-PyS.**

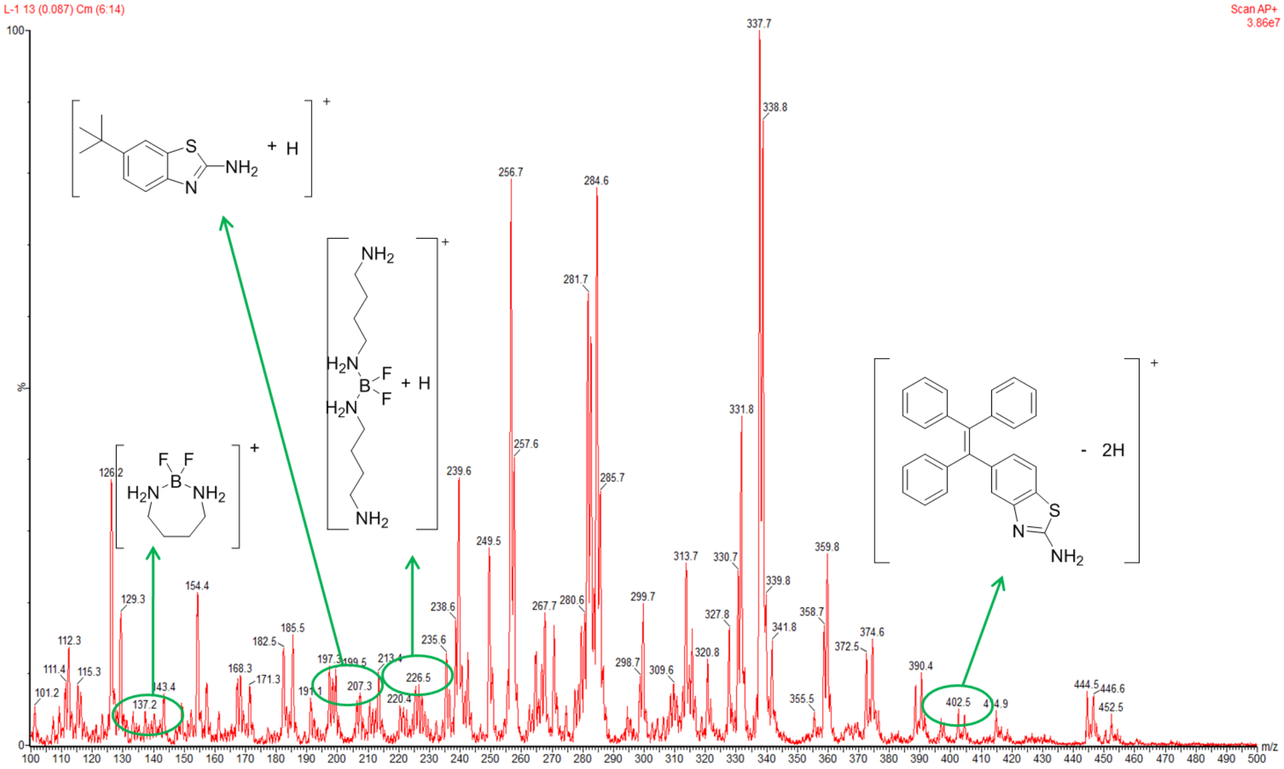

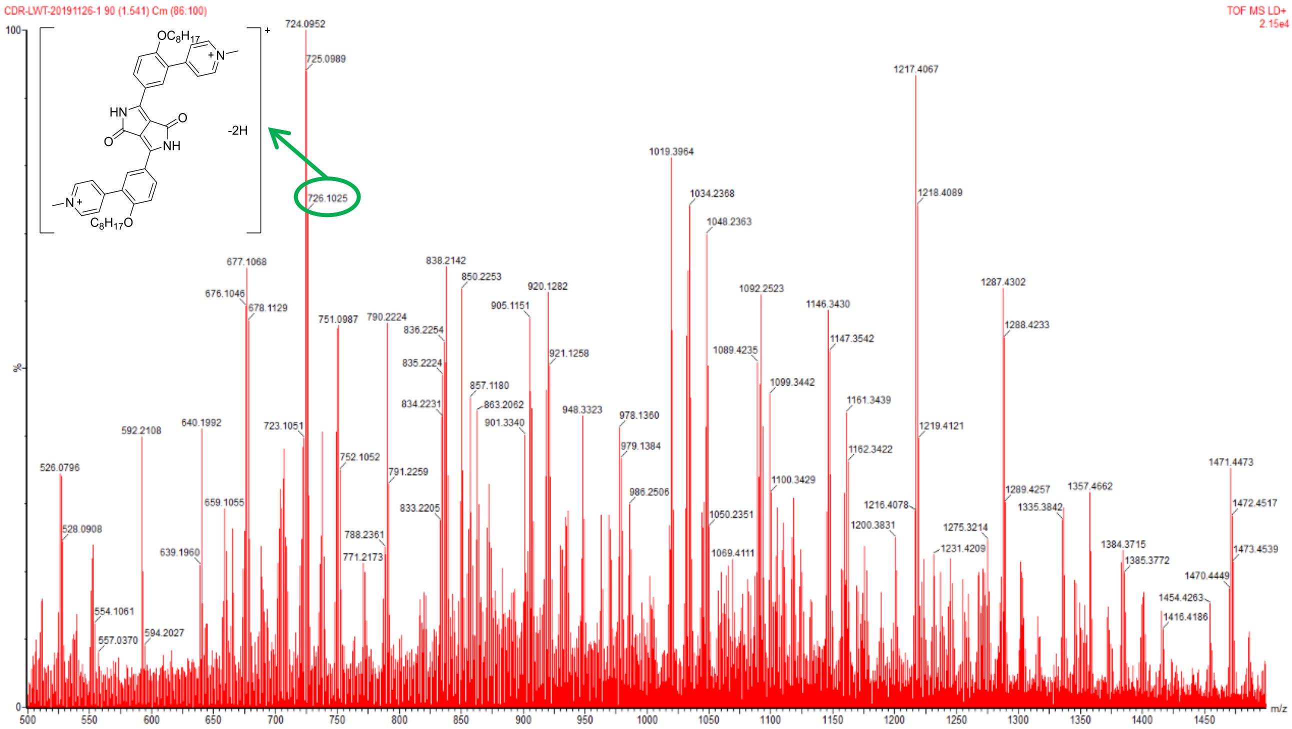

Supplementary Figure 12. The MS spectra of proposed reaction intermediates and products between **PPAB-PyS** and putrescine in MeCN.

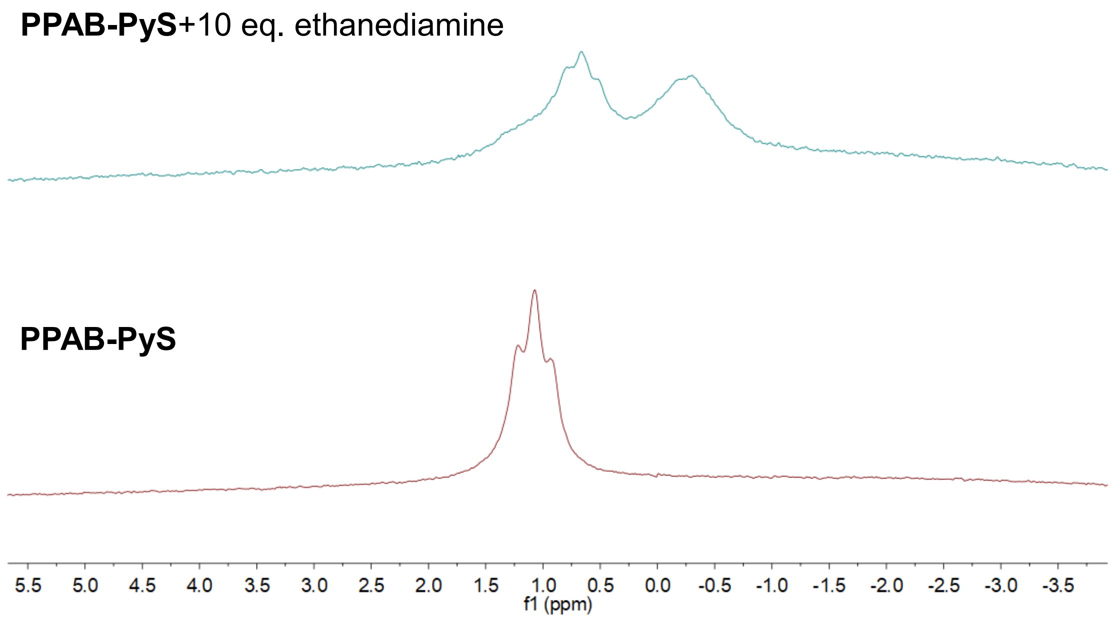

Supplementary Figure 13. ^{11}B NMR spectra of **PPAB-PyS**(10 mM) in absence and presence of putrescine (100 mM) in $CDCl_3$.

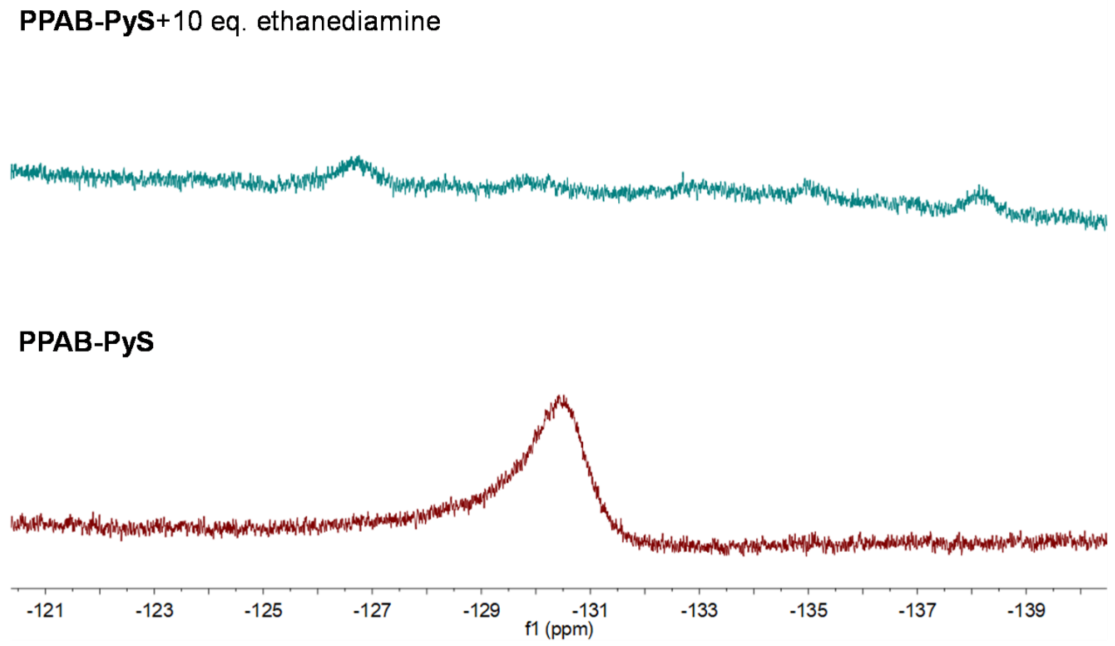

Supplementary Figure 14. ^{19}F NMR spectra of **PPAB-PyS**(10 mM) in absence and presence ofputrescine (100 mM) in $CDCl_3$.

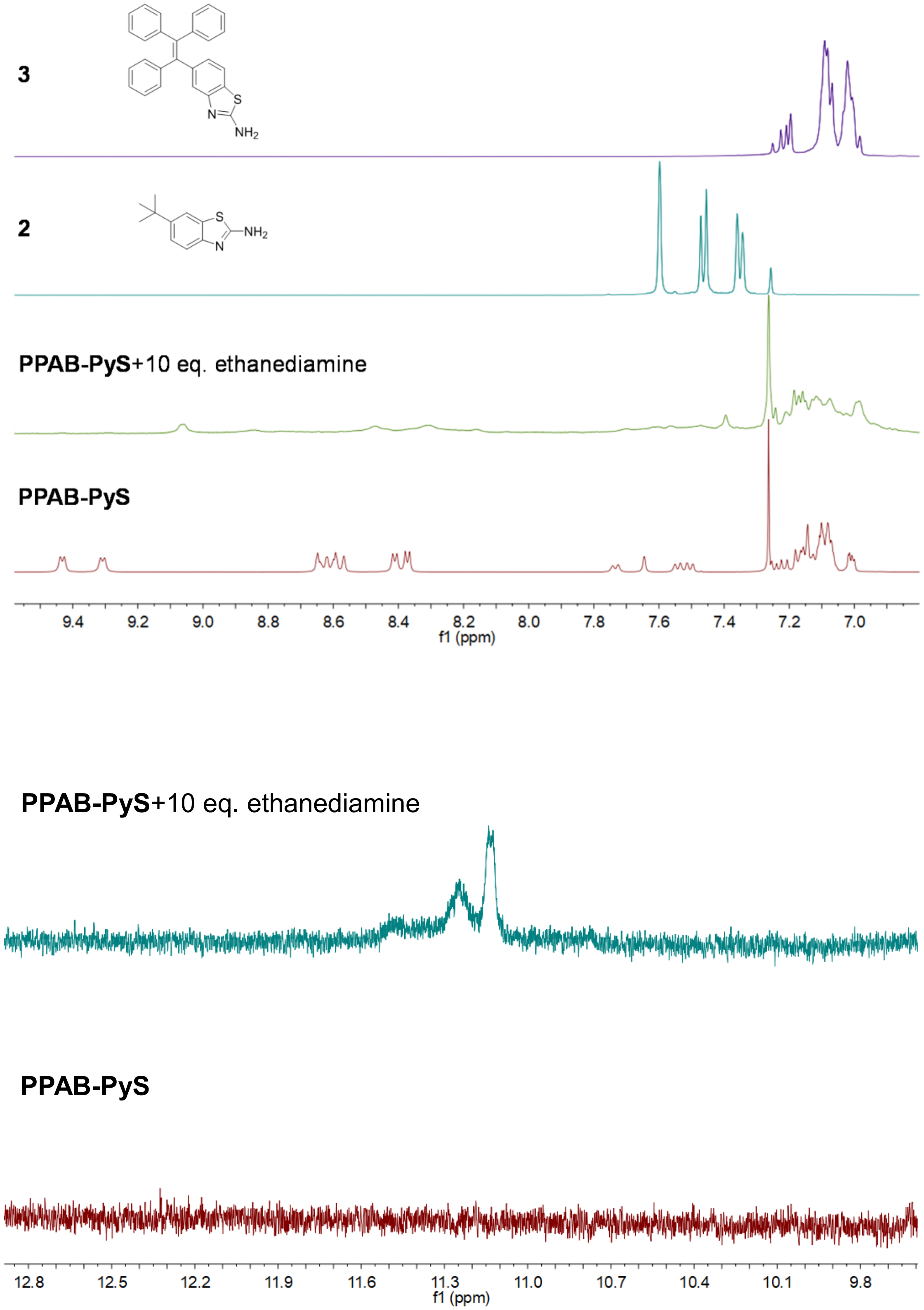

Supplementary Figure 15. ^{1}H NMR spectra of **PPAB-PyS** (10 mM) in absence and presence of ethanediamine (100 mM) in $CDCl_3$.

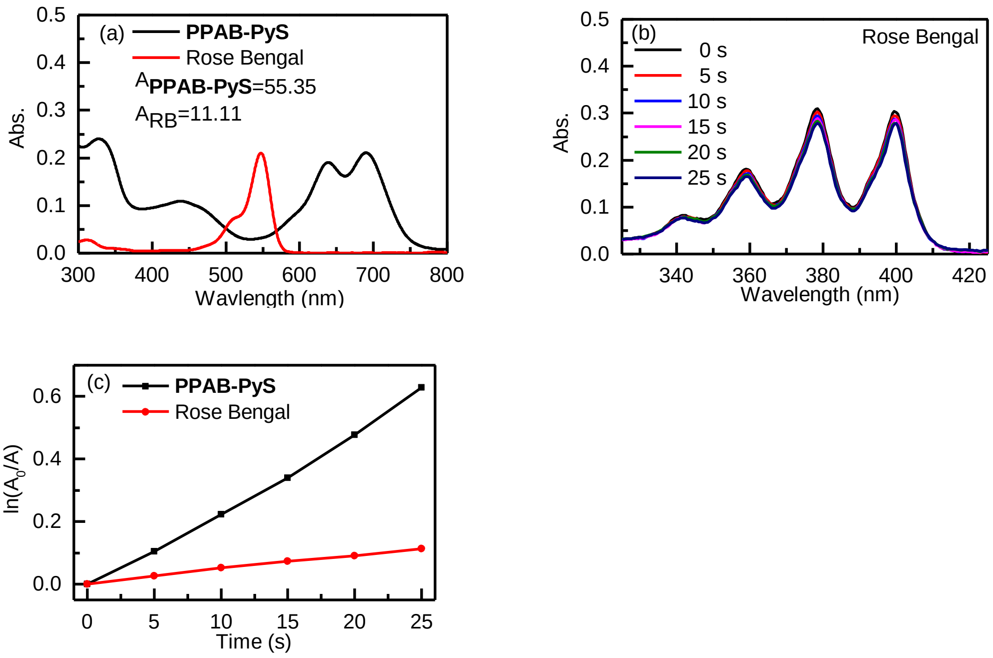

Supplementary Figure 16. (a) UV-Vis spectra of Rose Bengal (2μM)in DMSO/water (1/99, v/v)and **PPAB-PyS**(5 μM)in MeCN/water(1/99, v/v). (b) Time-dependent UV-Vis spectra of ABDA in presence of Rose Bengalin DMSO/water (1/99, v/v)under white light irradiation. (c) Decomposition rate of ABDA with **PPAB-PyS** and Rose Bengal, respectively. [ABDA] = 10×[PS], recording time interval: 5 s.

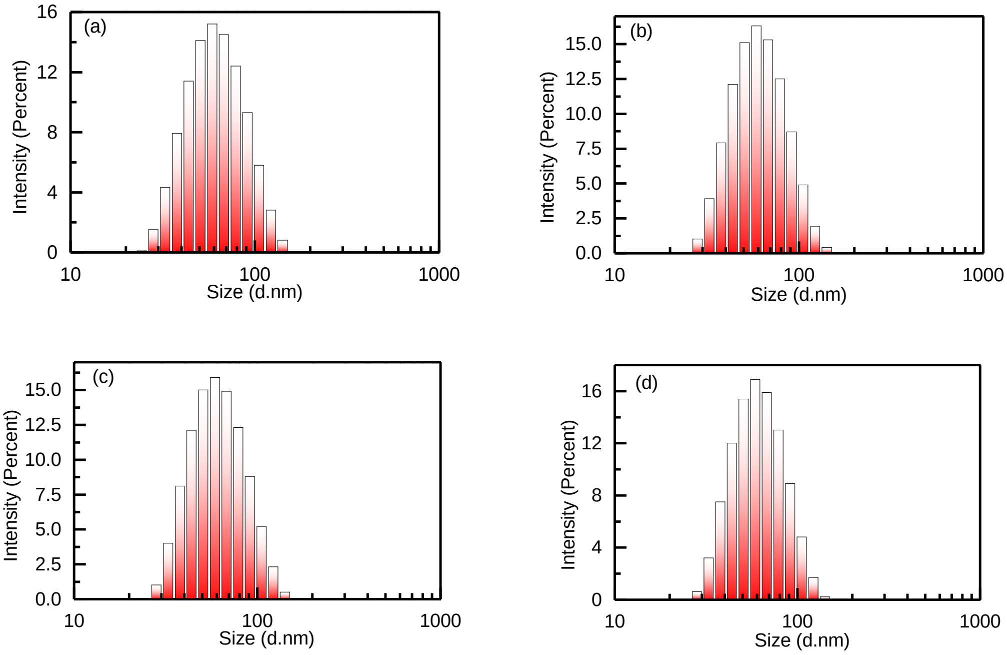

Supplementary Figure 17. The changes of hydrodynamic diameter of **PPAB-PyS** NPs at room temperature for (a) 0 h, (b)12 h, (c)48 h, (d) 96 h storage in water.

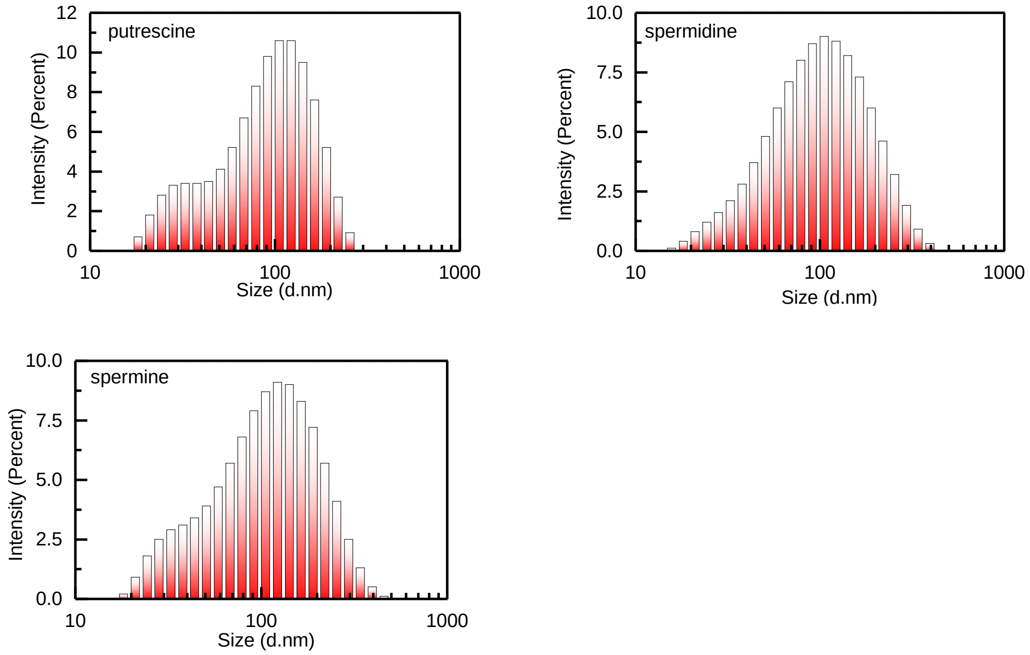

Supplementary Figure 18. The hydrodynamic diameter of **PPAB-PyS**NPs (10 μM) at room temperature in presence of putrescine, spermidine and spermine (40 equiv.).

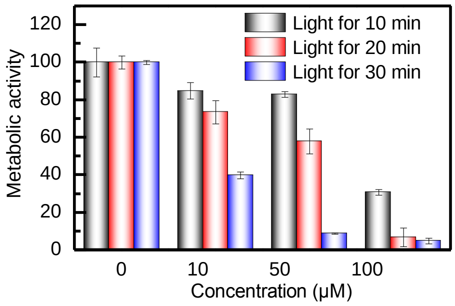

Supplementary Figure 19. Irradiation time dependent cell viability of HeLa cells stained with **PPAB-PyS** NPs.

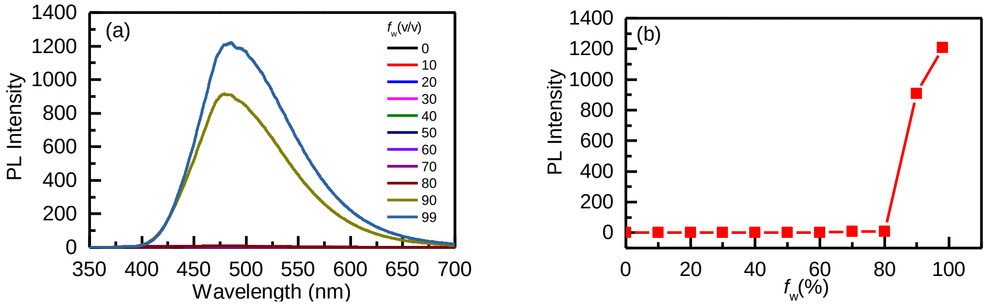

Supplementary Figure 20. (a) PL spectra of **3** in DMSO/water mixtures withdifferent water fractions (f_{w}). (b) Plot of the emission intensityversus f_w. Excitation wavelength: 380 nm; Concentration: 10 μM.

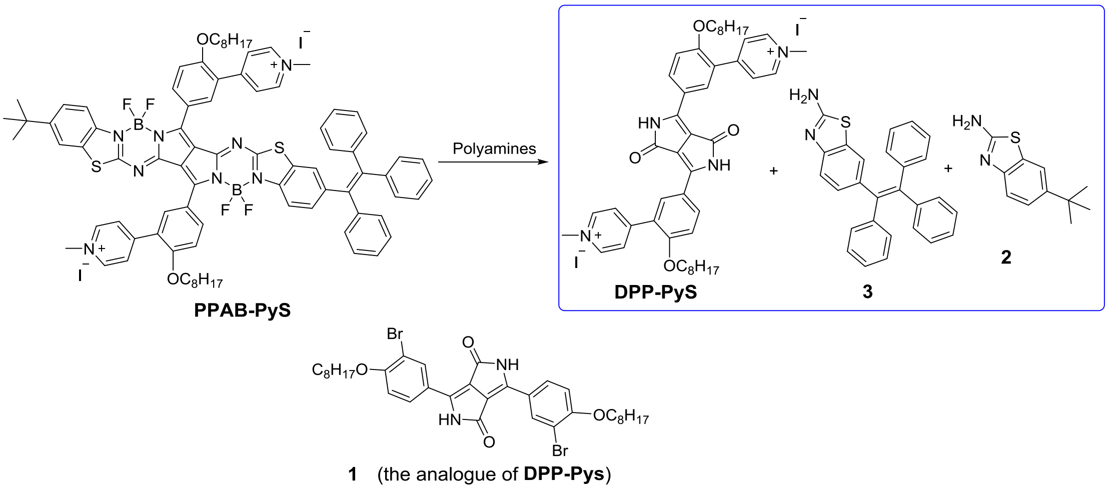

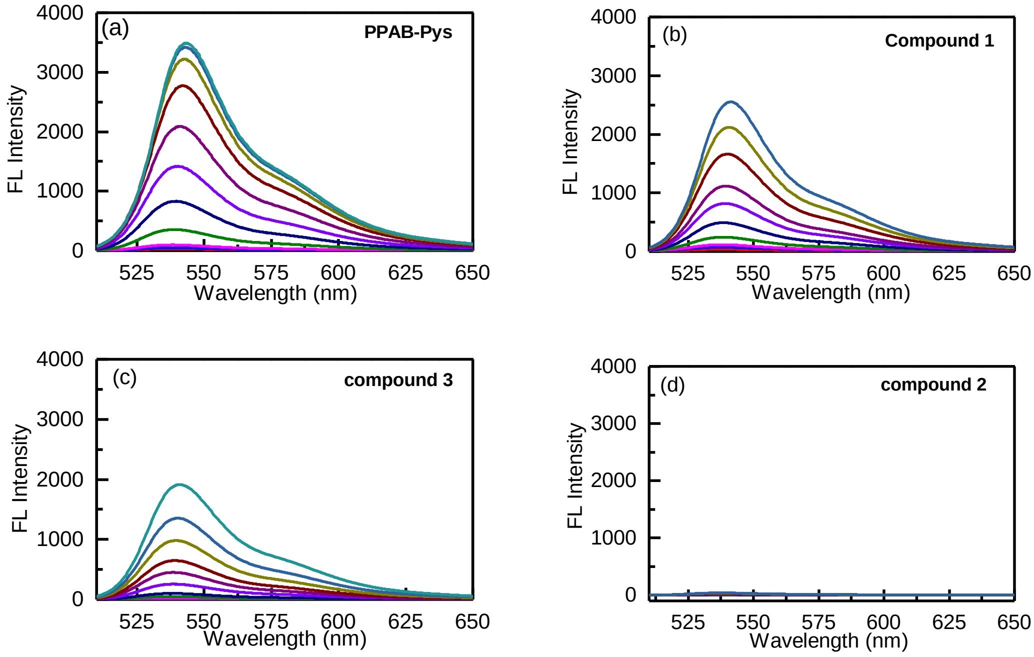

Supplementary Figure 21. Thefluorescencespectraof H2DCF-DA in the presence of (a)**PPAB-PyS**, (b)**1**, (c)**3**, (d)**2**, (e)PL intensity at 540 nm of four compoundsin MeCN upon white-light irradiation for different times. Concentration: 10 × 10^{-6} M (**PPAB-PyS**, **1**, **2** and **3**), 2× 10^{-4} M (H2DCF-DA); λ_{ex}: 488 nm.

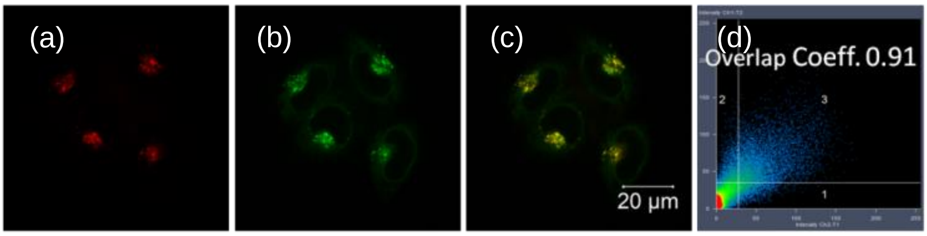

Supplementary Figure 22. Co-localization imaging of HeLa cells stained with (a) 100 μM **PPAB-PyS**NPs for 24h and (b) 100 nMLysoTracker® GreenDNDfor 30 min. (c) Merged images of a and b. (d) Scatter plot indicating the correction coefficientbetween a and b. For **PPAB-PyS**NPs in red channel: λ_{ex} = 633 nm, λ_{em} = 640–740 nm; LysoTracker® Green DND: λ_{ex} = 488 nm, λ_{em} = 493–550 nm.

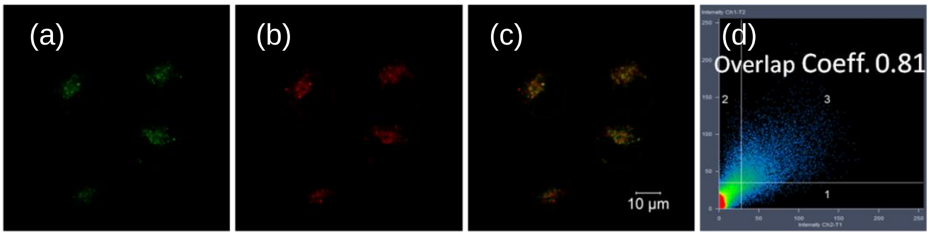

Supplementary Figure 23. Co-localization imaging of HeLa cells stained with(a) 10 μM **PPAB-PySNPs** for 24h and (b) 100 nMLyso-Tracker red DND-99 for 30 min. (c) Merged images of a and b. (d) Scatter plot indicating the correction coefficientbetween a and b. For **PPAB-PySNPs** in red channel: λ_{ex} = 405 nm,λ_{em} =500–580 nm;LysoTracker® Green DND:λ_{ex} = 543nm,λ_{em} = 590–610 nm.

The content of spermine in the sample was calculated according to the standard curve of polyamines (**Supplementary Figure 24**).The relative concentration of polyamines was determined by ratio between polyamine and protein concentration.

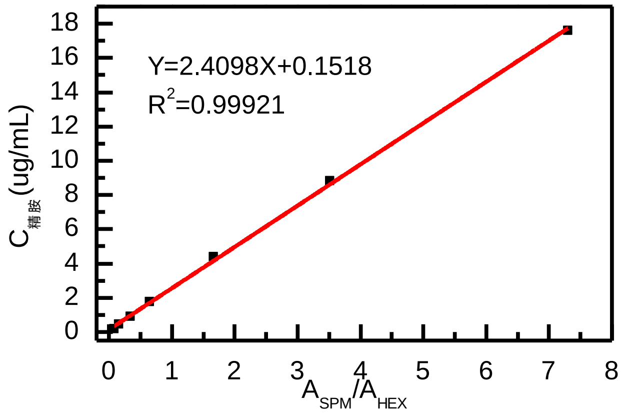

Supplementary Figure 24. Standard curve of spermine by HPLC.

Supplementary Table 1. Inhibitors that target polyamine metabolism, function and transport.

Inhibitors	Target	Structure	Status	Ref
DFMO	ODC	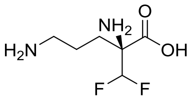	Approved for the treatment of Trypanosoma brucei subsp gambiense; Multiple ongoing clinical trials for cancer, including prostate cancer, lung cancer, and colon cancer.	1,2
BENSpm	SSATorSMO	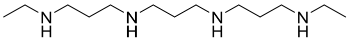	Preclinical use	3
CHENSpm	SSAT	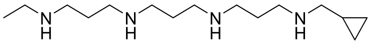	Preclinical use	4
F14512	DNA	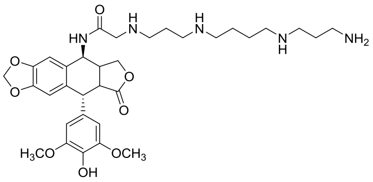	Clinical trials for the treatment of refractory/relapsing acute myeloid leukemia	5
PAP5A		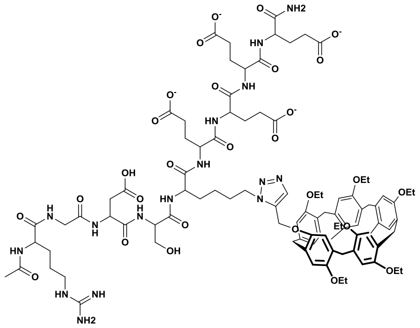	Experiments in vivo show that P1P5A effectively inhibits thegrowth of breast adenocarcinoma xenografts in female nude mice	6

Supplementary Table 2. The A_{350}/A_{670} and I_{430}/I_{710}multiplier for reaction between **PPAB-PyS** and amines.[a]

Entry	Amine	Enhanced multiple of A_{350}/A_{670}	Enhanced multiple of I_{430}/I_{710}
1	blank	1	1
2	spermine	167	20
3	spermidine	170	20
4	putrescine	60	10
5	ethanediamine	58	12
6	diaminopropane	66	20
7	cadaverine	46	11
8	n-propylamine	38	3
9	n-butylamine	35	3
10	n-hexylamine	44	6
11	cyclohexylamine	29	3
12	diethylamine	0.11	2
13	triethylamine	1	1
14	trimethylamine	1	3
15	phenylamine	1	1

[a]Reaction condition: **PPAB-PyS**(10 μM) in MeCN, amine (400 μM), 3 min.

Supplementary Table 3. The pseudo-first-order rate constants (k_{obs}) for the reaction between **PPAB-PyS**and amines.

Amines	Structure	pK_a	k_{obs} (*10^{-3} s^{-1})
spermine	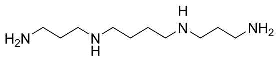	-	10.60
spermidine	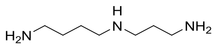	12.00	12.04
Putrescine	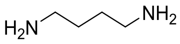	10.80	6.62
Ethylenediamine	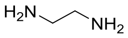	11.02	6.58
1,3-Propanediamine	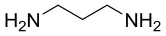	10.94	5.33
Cadaverine	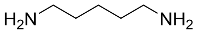	10.05	6.44
n-Hexylamine	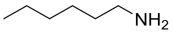	10.56	2.72
n-Propylamine	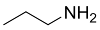	10.60	2.67
n-Butylamine	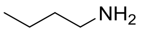	10.77	4.32

[a]pK_a data was from https://www.chemicalbook.com/. Reaction conditions: (1.25 μM) in MeCN in presence of amine (5μM) at 22 °C. The reaction rate of other amines with**PPAB-PyS** was too slow to obtain k_{obs} under the same test conditions.

Supplementary Table 4. The IC_{50} data for anticancer study based on polyamine depletion.

Entry	Agents	Cells	IC_{50}(μM)	Ref
1	oxaliplatin-carboxylatedpillar[6]arenehost-guestcomplex	HCT116	13.70	7
2	Peptide-pillar[5]arene conjugate	MCF-7	18.40	6
		C6	30.16	
		U87	22.53	
		A549	34.81	
		HepG-2	30.85	
		LO2	49.17	
		HUVEC	52.15	
3	**PPAB-PyS** NPs	HeLa	7.71	This work
		MCF-7	9.62	
		DU145	9.65	
		LO2	16.20	

References

1. M. D. Abeloff, S. T. Rosen, G. D. Luk, S. B. Baylin, M. Zeltzman, A. Sjoerdsma, *Cancer Treat. Rep*. **70**, 843–845 (1986)

2. Wallace HM, Fraser AV. *Biochem Soc Trans*, **31**,393-396 (2003).

3. Marverti G, Ligabue A, Paglietti G. *Eur. J. Pharmacol*, **615**, 17-26 (2009).

4.A. Kruczynski, A. Pillon, L. Creancier, I. Vandenberghe, B. Gomes, V. Brel, E. Fournier, J. P. Annereau, E. Currie, Y. Guminski, D. Bonnet, C. Bailly and N. Guilbaud, *Leukemia*, **27**, 2139–2148 (2013).

5. F. Mouawad, A. Gros, B. Rysman, C. Bal-Mahieu, C. Bertheau, S. Horn, T. Sarrazin, E. Lartigau, D. Chevalier, C. Bailly, A. Lansiaux and S. Meignan, *Oral Oncol*. **50**, 113–119 (2014).

6. Chen J, Ni H, Meng Z, Wang J, Huang X, Dong Y, Sun C, Zhang Y, Cui L, Li J, Jia X, Meng Q, *Nat Commun* **10**, 3546 (2019).

7. Hao Q, Chen Y, Huang Z, Xu J-F, Sun Z, Zhang X. *ACS Appl Mater Interfaces* **10**, 5365-5372 (2018).
