# Supplementary material for: Characterization of nanoparticles combining polyamine detection with photodynamic therapy
Source: Commun Biol. 2021 Jul 1;4:803. doi: 10.1038/s42003-021-02317-5 (PMC8249666; doi:10.1038/s42003-021-02317-5)
Supplement: Supplementary file 3 — Description of Supplementary Files [file 42003_2021_2317_MOESM3_ESM.pdf]

**Description of Additional Supplementary Files**

**File name:** Supplementary Data 1

**Description:** Original source data for Figures 2a, 2b,3a, 3b, 4b, 5b, 5d, 5f, 6b, 6d, 6f, 7c, 8, 9b, 9c, and 10, respectively.
